# Schizophrenia Patients Show Largely Similar Salience Signaling Compared to Healthy Controls in an Observational Task Environment

**DOI:** 10.3390/brainsci11121610

**Published:** 2021-12-06

**Authors:** Adam J. Culbreth, Zuzana Kasanova, Thomas J. Ross, Betty J. Salmeron, James M. Gold, Elliot A. Stein, James A. Waltz

**Affiliations:** 1 Maryland Psychiatric Research Center, Department of Psychiatry, University of Maryland School of Medicine, Baltimore, MD 21228, USA; JGold@som.umaryland.edu (J.M.G.); JWaltz@som.umaryland.edu (J.A.W.); 2 Leuven Research & Development Spin-off & Innovation Unit, KU Leuven, Waaistraat 6-Box 5105, 3000 Leuven, Belgium; zuzana.kasanova@kuleuven.be; 3 Neuroimaging Research Branch, National Institute on Drug Abuse-Intramural Research Program, Baltimore, MD 21224, USA; tross@intra.nida.nih.gov (T.J.R.); bsalmeron@intra.nida.nih.gov (B.J.S.); estein@intra.nida.nih.gov (E.A.S.)

**Keywords:** schizophrenia, dopamine, reinforcement, basal ganglia, ventromedial prefrontal cortex

## Abstract

Recent evidence suggests that the aberrant signaling of salience is associated with psychotic illness. Salience, however, can take many forms in task environments. For example, salience may refer to any of the following: (1) the valence of an outcome, (2) outcomes that are unexpected, called reward prediction errors (PEs), or (3) cues associated with uncertain outcomes. Here, we measure brain responses to different forms of salience in the context of a passive PE-signaling task, testing whether patients with schizophrenia (SZ) showed aberrant signaling of particular types of salience. We acquired event-related MRI data from 29 SZ patients and 23 controls during the performance of a passive outcome prediction task. Across groups, we found that the anterior insula and posterior parietal cortices were activated to multiple different types of salience, including PE magnitude and heightened levels of uncertainty. However, BOLD activation to salient events was not significantly different between patients and controls in many regions, including the insula, posterior parietal cortices, and default mode network nodes. Such results suggest that deficiencies in salience processing in SZ may not result from an impaired ability to signal salience *per se*, but instead the ability to use such signals to guide future actions. Notably, no between-group differences were observed in BOLD signal changes associated with PE-signaling in the striatum. However, positive symptom severity was found to significantly correlate with the magnitudes of salience contrasts in default mode network nodes. Our results suggest that, in an observational environment, SZ patients may show an intact ability to activate striatal and cortical regions to rewarding and non-rewarding salient events. Furthermore, reduced deactivation of a hypothesized default mode network node for SZ participants with high levels of positive symptoms, following salient events, point to abnormalities in interactions of the salience network with other brain networks, and their potential importance to positive symptoms.

## 1. Background

Motivated by the dopamine (DA) hypothesis of schizophrenia (SZ) and more recent formulations arguing that psychosis may emerge from faulty causal learning [1], brought on by frequent instances of aberrant salience signaling [2,3], there has been a tremendous increase in studies of DA and reward system signaling, associated with learning in SZ. Dopamine cells have been shown to play a critical role in feedback-driven learning, by signaling both the expectation of rewards and mismatches between expectations and outcomes, called prediction errors (PEs; [4]). Elegant experiments in nonhuman primates have demonstrated that positive PEs (better-than-expected outcomes) are signaled by phasic bursting of DA cells, while negative PEs (worse-than-expected outcomes) are signaled by phasic cessations of DA cell firing [5]. Functional imaging studies in human subjects have identified responses in the striatum that similarly signals the valence of *reward* PEs [6,7,8]. Importantly, there is now evidence that striatal PE signaling is disrupted in psychosis patients, most prominently in patients who are unmedicated [9], especially in the context of operant learning tasks [10]. Additional evidence points to aberrant unsigned (positive or negative) PE signals in prefrontal cortex (PFC) in both unmedicated [11] and medicated psychosis patients [12]. In these cases, unsigned PEs indicate the occurrence of a particular type of salient event, with the aberrant responses occurring in nodes of canonical brain networks for signaling salience—the anterior insula (AI), more specifically.

By contrast, we [13] had previously found that, although striatal responses to the delivery of a primary reinforcer (fruit juice) were disrupted in medicated SZ patients, striatal responses to the omission of an expected reward appeared intact. This finding of intact striatal responses to negative PEs has since been replicated by several groups [14,15,16]. This combination of findings points to several issues: (1) the importance of the medication status of patients in the sample; (2) the need to consider task-design factors, such as whether the task requires active acquisition of reward contingencies through classical or operant conditioning—or whether the contingencies had already been acquired—and neural responses to cues and outcomes were being evaluated without subject behavior being a key factor; and (3) the importance of considering striatal PE signals in the context of network interactions. Both signed and unsigned PEs are treated as salient events by the brain, evoking (likely downstream) responses in a variety of areas involved in the processing of salient events. Similar sets of areas are evoked by cues to uncertain/informative outcomes [17]. These areas include not only nodes of the brain’s salience network (SN), but also components of an executive control network (ECN), which has been shown to be activated by/in conjunction with the SN, and a default mode network (DMN), the activity of which is suppressed by networks involved in the processing of salient stimuli and the performance of cognitive tasks. Critically, a considerable literature points to reduced suppression of DMN activity in schizophrenia during task performance [18,19], suggesting a lack of inhibition from the SN and ECN [20]. Thus, even if SZ patients show intact activation of SN and ECN nodes—including the AI, the dorsomedial prefrontal cortex (DMPFC), and the posterior parietal cortex (PPC)—during outcome processing, they may still show reduced associated suppression of activity in DMN nodes, including the posterior cingulate cortex (PCC) and anterior medial PFC. In short, even if patients show intact outcome-related activation of AI and PPC, they may still show an aberrant salience response, in a larger sense.

In order to address these issues, we designed a passive, non-instrumental reward prediction task, in which participants were told that they were watching someone play a card game. Using this paradigm, we investigated brain responses to multiple kinds of salient events: cues highly predictive of outcome value, cues to uncertain outcomes, and signed and unsigned PEs. We sought to answer several specific questions:

1. Are the neural correlates of various kinds of salience, measured in a passive environment (where actions and outcomes are observed but have no impact on behavior), similar?
2. Is SZ associated with aberrant signaling of particular kinds of salience, and if so, is this reflected by brain regions within the SN or DMN?
3. Are the positive and negative symptoms of SZ associated with particular kinds of salience and associated neural correlates?

Given evidence that different types of PE signals have separable neural correlates, we hypothesized that particular types of salience would elicit separable patterns of BOLD activation. Specifically, we hypothesized that patients with SZ would show intact signaling of signed PEs in the striatum and intact signaling of unsigned PEs in the insula, consistent with prior observations in medicated SZ patient samples [15,16,21]. We also predicted that, in a passive environment, patients with SZ would show intact signaling of outcome uncertainty in the AI, DMPFC, and PPC. Finally, we predicted that, in a passive environment, patients with SZ would not show adaptive suppression of DMN structures (anterior medial PFC, e.g.,) in response to salient events.

## 2. Methods

### 2.1. Recruiting and Screening of Participants

A total of 29 SZ patients and 23 healthy control subjects, matched on demographic characteristics and smoking status, successfully completed the task in the MRI scanner. One participant with SZ was removed due to excessive head motion (see *MRI data acquisition*) All participants were right-handed, as determined by the Edinburgh Handedness Inventory [22], and provided written informed consent to protocols approved by the Institutional Review Boards of the National Institute on Drug Abuse’s Intramural Research Program (Protocol 05-DA-N401) and the University of Maryland School of Medicine (Protocol HP-00042701). To ensure understanding of the study, all participants with a diagnosis of SZ were administered the Evaluation to Sign Consent (ESC; [23]), a short questionnaire about study demands and risks, as well as subject rights. No patient was enrolled in the study without first demonstrating adequate performance (at least 10 points out of a possible 12) on the ESC. All patients were on stable antipsychotic medication regimens (no changes for four weeks), all with second-generation antipsychotics (SGAs). The diagnosis of SZ or schizoaffective disorder in patients was confirmed using the SCID-I [24], as was the absence of Axis I diagnoses in control participants. Control participants diagnosed with Axis II personality disorders (based on screening with the SIDP-R; [25]) were also excluded. All participants underwent medical screening, involving a medical history and physical exam. Exclusionary criteria included: pregnancy, current illegal drug use (both verified by urine screens), admission of past substance dependence, and any neurological or medical illness that might confound data interpretation. Participants were instructed to abstain from alcohol for 24 h prior to study visits (verified by a breathalyzer); smokers were allowed to smoke prior to the MRI scanning, so as to avoid potential effects of nicotine withdrawal. See Table 1 for descriptive and characterizing data.

### 2.2. General Procedures 

Outside of the MRI scanner, cognitive function was assessed using three standard measures: the Wechsler Abbreviated Scale of Intelligence (WASI; [26]), the Wechsler Test of Adult Reading (WTAR; [27]), and the Repeatable Battery for the Assessment of Neuropsychological Status (RBANS; [28,29]). In order to assess the extent to which all study participants experience pleasure both physically and in social contexts, all subjects completed the Scales for Physical and Social Anhedonia [30]. Standard symptom ratings were obtained for all patients using the Scale for the Assessment of Negative Symptoms (SANS; [31]) the Brief Psychiatric Rating Scale (BPRS; [32]), and the Calgary Depression Scale (CDS; [33]).

For negative symptom severity, we averaged all items from the SANS to create a total score. For positive symptom severity, we averaged BPRS items including suspiciousness, grandiosity, hallucinations, and unusual thought content, to create a reality distortion subscale.

### 2.3. Outcome Prediction Task (OPT)

**Figure 1:**
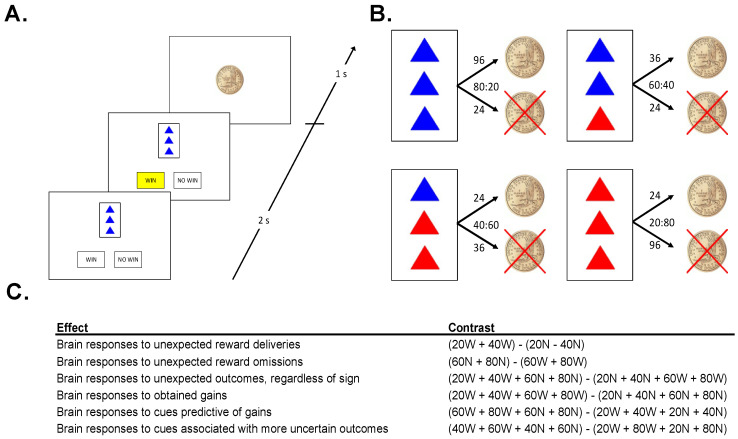
(**A**). *Sample Trial:* On each trial, participants were presented with 1 of 4 cues, then predicted the outcome of the cue (“WIN” or “NO WIN”) and were shown an outcome. (**B**). *Cues*: The task consisted of 4 different cues. Numbers above arrows indicate the number of trials that the cue resulted in each outcome, ratios denote win probabilities (WIN:NO WIN). (**C**). *Neuroimaging Contrasts*: five contrasts were analyzed to examine different types of salience.

In order to investigate processes involved in the expectation and receipt or omission of monetary rewards, we administered a passive task designed to evoke PEs, in conjunction with functional magnetic resonance imaging (fMRI; see Figure 1A). Participants were given the following instructions:

This experiment is a little like watching someone play a casino game. Since you are just watching, you cannot influence the outcome of the game at all. The person playing the game (whom you cannot see) is dealt one card at a time. That card either wins the player a chip, or not.

On each trial, 1 of 4 cards was displayed for 2 s. Each card had 3 triangles on it, colored either red or blue, followed by a prompt, asking the subject whether he/she expected the cue to be followed by a coin, or not. Once the subject made the prediction, the choice (“WIN” or “NO WIN”) was highlighted in yellow, until the end of the 2 s response window (Figure 1A). The offset of the card and the highlighted prediction was followed by a fixation of 500 ms, and then the 1 s presentation of the outcome: either a gold dollar coin (indicating the gain of a coin), or a gold dollar coin with an “X” through it (indicating the failure to win a coin). Each card predicted the gain or a no gain with a given probability (Figure 1B). The presentation of the outcome was followed by a fixation interval selected pseudo-randomly from a list (mean: 2500 ms; range: 500–4500 ms, step 500 ms). Importantly, in this observational task environment outcomes were (1) not able to be influenced by participants, and (2) not personally relevant for participants.

No stimulus was associated with exclusively positive, or exclusively negative, feedback (Figure 1B). However, the more blue triangles a card had, the more likely it was to win a coin. A card with 3 blue triangles was followed by a gain on 80% of trials and a no win on 20% of trials. A card with 2 blue triangles was followed by a gain on 60% of trials and a no win on 40% of trials. Cards with 1 and 0 blue triangles led to coins on 40% and 20% of trials, respectively. Participants completed 4 runs of the task in the MRI scanner, each lasting approximately 8.5 min, each involving the presentation of 90 trials. Following each run, participants were prompted to use an analog scale to estimate how frequently a stimulus is followed by the gain of a coin. This was done to verify that participants had a relatively accurate representation of the contingencies and were engaged in the task.

Because the purpose of the experiment was to investigate brain activity evoked by mismatches between expectation and outcomes, and not to assess brain activity during the acquisition of probabilistic contingencies, participants were not explicitly told the reinforcement contingencies of the task. Rather, participants were able to familiarize themselves with the task and acquire the reinforcement contingencies in practice sessions on the bench (60 observation trials, 60 prediction trials) and in a mock scanner (90 prediction trials), with the same contingencies as in the MRI task.

### 2.4. MRI Data Acquisition

Following these practice sessions (on the same day), participants underwent MRI scanning. In conjunction with the performance of the outcome prediction task, whole-brain functional EPI images were acquired using a 3-T Siemens Allegra scanner (Erlangen, Germany) for measurement of T2*-weighted BOLD effects (64 × 64 matrix; FOV = 22 × 22 cm; TR = 2 s; TE = 27 ms; FA = 80°). In order to reduce susceptibility artifacts [34], we used 4 mm oblique axial slices, 30° axial to coronal. We acquired 258 volumes in each of the 4 runs, with each run consisting of 90 trials averaging 5.5 s in length. The 4 EPI scanning runs thus lasted a total of 34.5 min and included 360 task trials. Head motion was minimized using foam padding or foam molding, and in each scanning session, a whole-brain oblique axial T1-weighted structural image (MPRAGE) was acquired for anatomical reference (1 mm^3^ isotropic voxels; TR = 2.5 s; TE = 4.38 ms; FA = 8°). One patient was removed from subsequent analyses due to excessive head motion (>3 mm in any direction).

### 2.5. Analyses of Behavioral Data

Among the 28 patients and 23 controls remaining (see Table 1 for characterizing data), we examined 2 aspects of behavior, in order to ascertain if participants had acquired the probabilistic contingencies: their within-trial predictions of outcomes (i.e., whether they believed the cue would be followed by a coin or not), and their between-run estimations of the reinforcement probability associated with each card (i.e., analog scale estimates of the frequency of a cue being followed by the gain of a coin). In each case, we recorded values for each cue/card for each participant, both the percentage of trials on which a reward was predicted for a particular cue, and the average of reinforcement probability estimates from the rating sessions following each of the four runs. We performed repeated-measures analyses of variance (ANOVAs) to assess the effects of diagnostic group and cue/card on both the percentage of trials on which a reward was predicted for a particular coin, and the average of reinforcement probability estimates from the rating sessions following each of the four runs.

### 2.6. Preprocessing of Event-Related Data

All preprocessing, first-, and second-level analyses of MRI data were performed using the AFNI software package [35]. Preprocessing steps included volume-registration for motion correction, slice-timing correction, temporal normalization, and blurring to a full-width, with a half-maximum of 8 mm. Regressors in general linear models (GLMs) of single-subject time series included the eight different trial types (four different cues × two possible outcomes). Regressors were three-second boxcar functions, time-locked to the onset of individual trials and convolved with a model hemodynamic response function (HRF). To help account for residual motion effects, head-motion curves were included in each GLM as regressors of no interest.

### 2.7. BOLD Signal Contrasts

We computed specific contrasts in a set of a priori regions of interest (ROIs) (Figure 1C). Specifically, each trial was classified according to its PE magnitude (high vs. low), PE valence (positive vs. negative), reward omission likelihood (large vs. small), and outcome certainty (large vs. small). In order to assess brain responses to unexpected outcomes regardless of valence (PE magnitude effect), we contrasted trials on which cues were followed by more probable outcomes (20% and 40% reward cues not followed by rewards, 60% and 80% reward cues followed by rewards) with trials on which cues were followed by less probable outcomes (20% and 40% reward cues followed by rewards, 60% and 80% reward cues not followed by rewards). In order to assess brain responses to obtained gains (PE valence effect), we contrasted trials involving rewarding outcomes with those involving non-rewarding outcomes. In order to assess brain responses to cues predictive of no gain (reward omission likelihood effect), we contrasted cues predictive of non-wins with cues predictive of wins (regardless of outcome). In order to assess brain responses to cues associated with more uncertain outcomes (certainty effect), we contrasted trials involving 40% and 60% reward cues (regardless of outcome) with trials involving 20% and 80% reward cues (regardless of outcome). Finally, for VS ROIs we analyzed brain responses to both unexpected reward deliveries and unexpected reward omissions (i.e., positive and negative prediction errors). Table S1 summarizes each of these contrasts. Contrasts are also listed in Figure 1B.

### 2.8. ROI Analyses

Coordinates of ROIs involved in the signaling of salience were drawn from the results of several studies upon which the current investigation was based. Specifically, investigation of feedback-evoked responses in the ventral striatum was motivated by numerous findings linking VS activity to the signaling of reward PEs [8,36]. We used spheres of 5 mm radius in left and right VS, using coordinates (+10, 8, −4) from the original study of reward-PE-signaling by Pessiglione et al. (2006). Coordinates for anterior insula (bilaterally) and posterior parietal cortex (bilaterally) were drawn from Huettel et al. (2005). Finally, coordinates of DMN nodes were taken from analyses of resting-state functional connectivity (rsFC) MRI data, acquired in the same scanning sessions as the event-related data for this study, by placing a seed in the posterior parietal cortex [21]. Coordinates for these ROIs are listed in the Supplementary materials (Supplementary Table S2).

### 2.9. Associations with Symptoms

In order to examine how symptom severity modulated feedback-related responses, we performed correlation analyses, involving contrasts between neural responses and different types of feedback events in the same set of brain regions described above. Pearson correlation analyses assessed relationships among negative symptom severity in SZ patients, positive symptom severity (BPRS reality distortion), and BOLD signal contrasts in the above-mentioned ROIs. We computed the BPRS reality distortion subscale by averaging item scores for suspiciousness, grandiosity, hallucinations, and unusual thought content.

### 2.10. Supplementary Analysis in a Reduced Sample

We conducted supplementary analyses of the MRI data to rule out a general performance deficit as an explanation for differences in brain activations. Thus, in a second set of group-level analyses of MRI data, identical to the analyses mentioned above, we included only participants who demonstrated sufficient acquisition of the relative reinforcement probabilities of the four cards. Subjects whose average between-run reinforcement-frequency estimates were not within 20% of actual contingencies were excluded. These criteria resulted in the removal of 11 patients and 3 controls from the initial analyses, leaving 17 patients and 20 controls. Characterizing information for participants included in these analyses is shown in Supplementary Table S3.

## 3. Results

### 3.1. Behavioral Data

**Figure 2:**
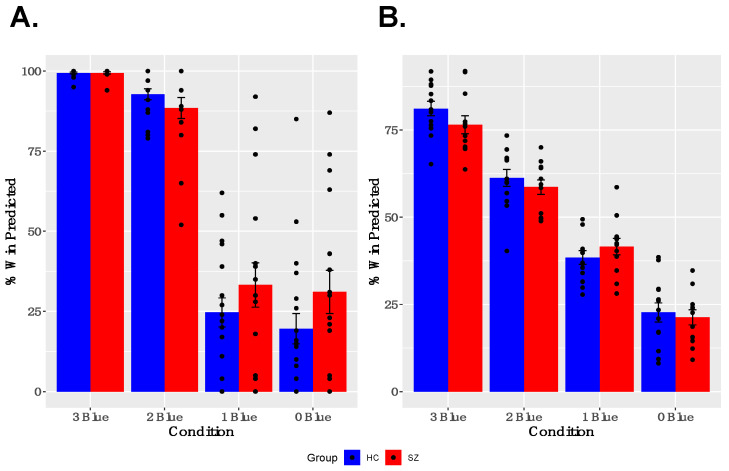
*Behavior Results:* (**A**) Participants modulated their trial-by-trial predictions of winning, based on the reward probabilities associated with each cue. (**B**) Participants successfully estimated reward probabilities associated with each cue at the between neuroimaging task runs.

As shown in Figure 2A,B, both groups showed learning of reward probabilities and sensitivity to contingencies associated with particular cues. Specifically, participants modulated their trial-wise predictions of winning, given the reward probabilities associated with particular cues, and gave appropriate estimates of the reward probabilities for particular cues when asked between runs. A two-way ANOVA for trial-wise reward predictions, with factors of GROUP and CUE, revealed a significant main effect of CUE [F(3,47) = 180.635, *p* < 0.001] and a significant GROUP × CUE interaction [F(3,47) = 5.443, *p* = 0.001], but no main effect of GROUP [F(1,49) = 1.918, *p* = 0.172]. The interaction was driven by a tendency for SZ participants to predict both a higher likelihood of winning for cues associated with infrequent winning and a lower likelihood of winning for cues associated with frequent winning, compared with healthy controls. The two-way ANOVA for estimated reward frequency after each run, with factors of GROUP and CUE, revealed a significant main effect of CUE [F(3,35) = 96.860, *p* < 0.001], no significant GROUP × CUE interaction [F(3,35) = 2.130, *p* = 0.100], and no main effect of GROUP [F(1,37) = 0.423, *p* = 0.519]. Both the reduced and full samples showed robust effects of CUE; however, the small effects of GROUP and GROUP × CUE interactions described above were not found in the reduced sample (See Supplementary Materials Figure S1 for description of behavioral performance in the reduced sample).

### 3.2. ROI Analyses

**Figure 3:**
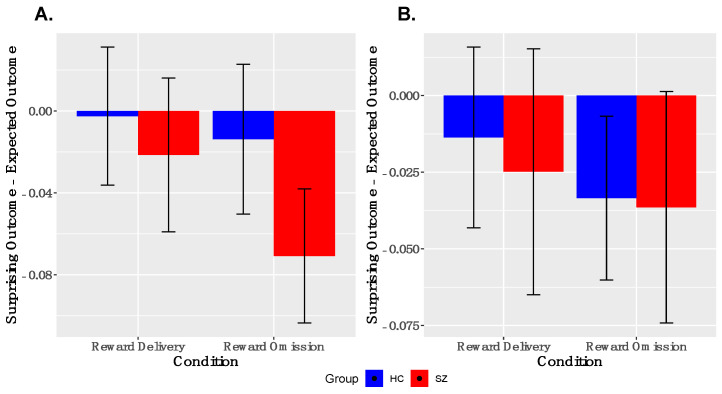
*Ventral Striatal Signaling to Prediction Error:* ANOVA responses to surprising reward omission, but not surprising reward deliveries, is evidenced in both left, (**A**), and right, (**B**) ventral striatum (y = 8). No between-group differences were observed. SZ, schizophrenia; HC, healthy control.

*Analyses of PE-signaling in ventral striatum*. As shown in Figure 3, we observed robust deactivations to unexpected reward omissions in the entire sample in both left and right VS; the groups did not differ in the magnitudes of these deactivations in either left or right VS. We did not observe robust activations to unexpected reward deliveries in the entire sample in either left or right VS. These findings were similar when examined within the reduced sample (Supplementary Figure S2).

**Figure 4:**
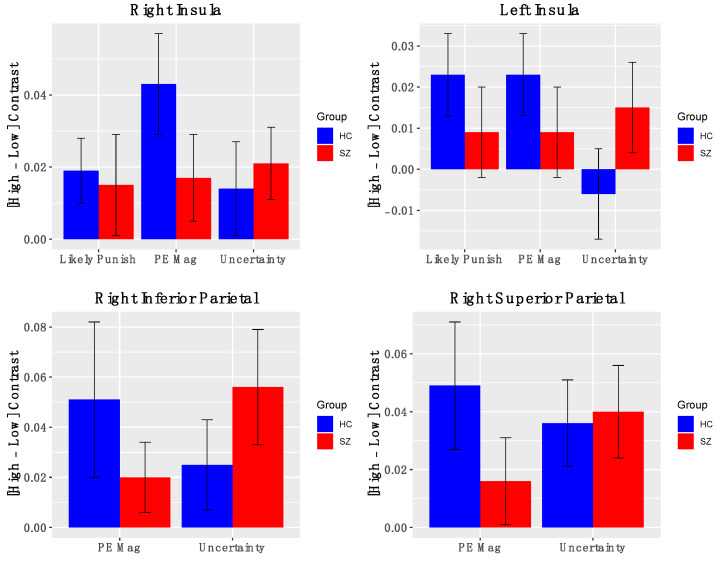
*BOLD activation of Salience Nodes to fMRI contrasts*: Error bars represent standard error of the mean; HC—healthy control; SZ—schizophrenia.

*Salience Network Nodes*. Across all participants, the AI showed significant positive activation for contrasts indexing PE magnitude (Figure 4) (left AI: t(50) = 2.03, *p* = 0.04; right AI: t(50) = 3.14, *p* = 0.003), levels of uncertainty (left AI: t(50) = 0.67, *p* = 0.49; right AI: t(50) = 2.19, *p* = 0.03), and likelihood of reward omission (left AI: t(50) = 2.03, *p* = 0.047; right AI: t(50) = 1.88, *p* = 0.07). Similarly, significant activations were found for right inferior and superior parietal cortical ROIs for contrasts indexing PE magnitude (right superior parietal: t(50) = 2.2, *p* = 0.04; right inferior parietal: t(50) = 2.4, *p* = 0.02) and heightened uncertainty (right superior parietal: t(50) = 2.8, *p* = 0.006; right inferior parietal: t(50) = 3.5, *p* = 0.001). Notably, BOLD activations for these salience contrasts did not significantly differ between groups. Effects were similar for the full and reduced samples (Supplementary Materials, Figure S3). Thus, we observed evidence that the bilateral insula and right posterior parietal cortex are signaling multiple types of salience.

**Figure 5:**
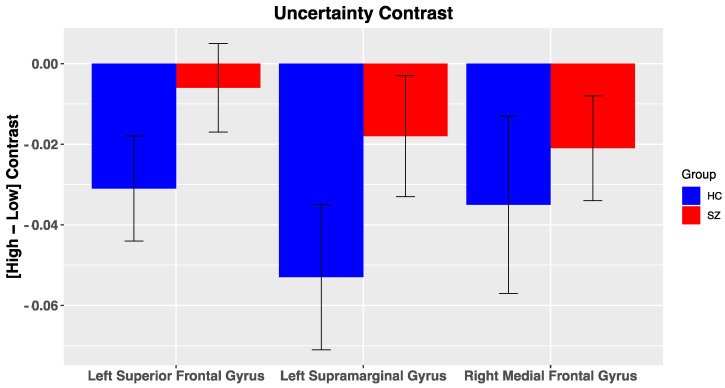
*BOLD activation of Default Mode Nodes to fMRI contrasts*: Error bars represent standard error of the mean; HC, healthy control; SZ, schizophrenia.

*DMN Nodes*. Across all participants, multiple ROIs within the DMN were reliably deactivated for the uncertainty contrast, including the left supramarginal gyrus (Figure 5; t(50) = −2.9, *p* = 0.005, right medial frontal gyrus (t(50) = −2.2, *p* = 0.03), and left superior frontal gyrus (t(50) = −2.05, *p* = 0.046). No other significant effects were noted within the DMN for the other salience contrasts (i.e., PE valence, PE magnitude, likely reward omission). Furthermore, we did not observe significant differences in BOLD activation of DMN nodes to our various salience contrasts between groups, potentially due to high variability in BOLD activation. In the reduced sample, group differences for DMN nodes were in a similar direction but did not reach significance (Supplementary Materials, Figure S4).

**Figure 6:**
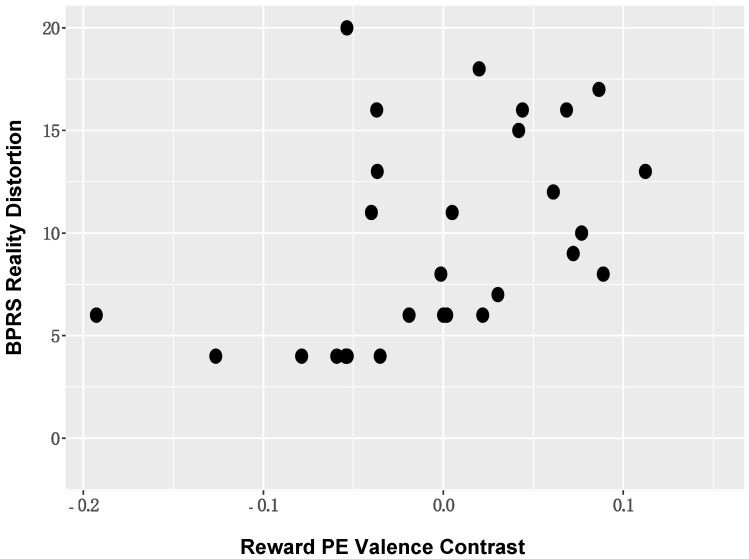
*Positive Symptom Severity and DMN BOLD Activation:* Positive symptom severity (BPRS reality distortion) in those with schizophrenia was positively associated with BOLD activation in DMN nodes, including the left supramarginal gyrus.

*Correlation analyses between ROI activation and symptom severity*. When considering the BOLD activation to PE valence, positive symptom severity (BPRS reality distortion) in those with SZ was positively associated with BOLD activation in the left supramarginal gyrus (r = 0.43, *p* = 0.02; Figure 6). The direction of this association suggested that individuals with more severe positive symptoms showed reduced deactivation of DMN nodes. These associations remained significant in the reduced sample (Supplementary Materials Figures S5–S9). No other significant symptom relationships were identified.

## 4. Discussion

Our results indicate that, in an observational environment, where individuals are unable to influence outcomes through their behavior and the outcomes are not personally relevant, the anterior insula and posterior parietal cortices were significantly activated to multiple different types of salience, including PE magnitude, likelihood of reward omission, and heightened levels of uncertainty. Surprisingly, patients with SZ showed largely similar BOLD activation to controls, suggesting potentially intact ability to activate striatal and cortical regions involved in the signaling of rewarding salient events. For example, SZ patients did not differ from controls in their deactivations of ventral striatum in response to unexpected reward omissions. However, we observed a positive association between the severity of positive symptoms and the magnitudes of salience contrasts in DMN node (e.g., supramarginal gyrus). Most reported effects either remained significant or were in a similar direction for both the full and reduced samples; this consistency suggests that a generalized performance deficit on the task does not account for the observed findings. Taken together, the current study provides preliminary evidence of similarities and differences in salience signaling between healthy controls and people with SZ in an observational context, where participants are both unable to influence outcomes and outcomes are not personally relevant for the participant.

Our findings raise several questions about the role of the striatum in PE signaling and its possible disruption in SZ. Our finding of relatively intact striatal PE signals in medicated SZ patients, performing a passive task, contrasts with findings of disrupted striatal PE signals in unmedicated SZ patients, performing operant tasks [9,37]. This may be explained by either the medication status of our patients, the passive nature of the task, or a combination of both. In fact, the current results fit with several previous findings of intact reward-related striatal signaling in medicated SZ patients, especially with regard to the signaling of negative reward PEs [13,15,16]. There is additional evidence that second-generation antipsychotics may “normalize” striatal reward signals in SZ [12,38], in contrast to first-generation antipsychotics [39]. However, work from the basic cognitive neuroscience literature also suggests that striatal involvement in tasks of learning and motivation may be more related to the anticipation of action than the anticipation of outcomes [40], and that PE signaling in the context of a passive task may be less reliant on the striatum [41]. Importantly, in operant tasks, outcomes are typically (1) able to be influenced by participants, and (2) personally relevant for participants. Thus, our task where a subjects’ actions have no influence on outcomes may evoke a different kind of striatal reinforcement learning signal from that observed in the context of an operant task. It may be that the striatal signals that are most disrupted in SZ (and are most closely related to motivational deficits in SZ) are those involved in signaling prospective actions, and the expected consequences of prospective actions. Furthermore, it may be that there is relatively smaller reinforcement in the current design, as individuals are not actually receiving rewards.

The current results also raise several questions about the role of the anterior insula and posterior parietal cortex in salience signaling and its supposed aberrance in SZ. Specifically, we did not find significant between-group differences in these regions. However, it should be noted that most, if not all, of schizophrenia vs. control between-group differences have emerged from findings using operant tasks. We argue that VS and AI responses to salient events in SZ patients may be particularly disrupted when events have motivational salience (i.e., implications for behavioral modification), which may explain the lack of between-group differences in the current design. Broadly, this set of findings is consistent with the adjacent literature characterizing hedonic capacity in SZ, which finds that individuals with SZ show intact experience of rewards in the moment, but show difficulties in using rewards to guide future decision making and behavior [42,43,44]. 

Finally, while we did not observe between-group difference in DMN deactivations, our result—demonstrating a positive association between reduced DMN suppression and positive symptom severity—is consistent with several previous reports. Broadly, such findings are consistent with the seminal work of Kapur and colleagues [3], suggesting that aberrant salience could lead to the formation and maintenance of delusions. Furthermore, numerous studies have pointed to a role for DMN suppression in attentional processes [18,45]. Previous results from our group [21] have indicated that DMN suppression during outcome processing is critical for successful reinforcement learning. The current results further support the idea that a reduced ability to suppress the DMN during task performance, in general, and outcome processing, in particular, is characteristic of patients with SZ with higher levels of positive symptoms.

Our ability to generalize the results of this study is limited by several factors. First, the size of our final analysis sample was small. Second, we are unable to draw definitive conclusions about the impact of antipsychotic medications in our study, as all patient participants were medicated and clinically-stable. However, a thorough assessment of the impact of antipsychotic medications on salience signaling in a reward prediction task would require random assignment to drug, in the context of a controlled clinical trial.

In summary, the results of the current study extend the previous literature on salience signaling in SZ by reporting on neural response to different types of salient events in an observational environment. Across participants, we found that the anterior insula and posterior parietal cortices were reliably activated to multiple different types of salience, including prediction error magnitude, likelihood of reward omission, and heightened levels of uncertainty. Thus, our results suggest that, in an observational environment, SZ patients may show an intact ability to activate striatal and cortical regions to rewarding and non-rewarding salient events. When coupled with previous work, showing disrupted salience signaling in SZ when events have motivational salience (i.e., implications for future action), our results suggest that people with SZ may have intact experience of salience but diminished ability in using such signals to drive future behavior. Furthermore, while we failed to demonstrate robust between-group differences in salience signaling between groups, positive symptom severity was found to significantly correlate with the magnitudes of salience contrasts in DMN nodes. This result further supports the idea that a reduced ability to suppress the DMN during task performance, in general, and outcome processing, in particular, is characteristic of patients with schizophrenia with higher levels of positive symptoms.

## Figures and Tables

**Table 1:** Subject characterizing information.

	SZ Patients	(*n* = 28)	Controls	(*n* = 23)	Sig. of Group Diff.
*Demographics*					
Age	40.3	(10.0)	41.3	(10.8)	*p* = 0.750
Gender	4 F,	24 M	6 F,	17 M	*p* = 0.316
Race	19 W,	9 NW	16 W,	7 NW	*p* = 1.000
Smokers	10 Y,	18 N	5 Y,	18 N	*p* = 0.360
Subject Education (years)	13.4	(1.7)	15.1	(2.1)	***p* = 0.002**
Parental Education (years)	13.9	(2.8)	13.8	(2.4)	*p* = 0.863
*Neuropsychological Testing/* *Questionnaires*					
IQ (from WASI 4-subtest)	101.2	(15.0)	115.4	(12.0)	***p* = 0.001**
WTAR Scaled Score	100.6	(17.8)	109.1	(11.8)	***p* = 0.046**
RBANS Total	85.3	(16.5)	102.3	(10.2)	***p* < 0.001**
Chapman—Phys. Anhed.	15.4	(9.5)	10.6	(9.1)	*p* = 0.077
Chapman—Soc. Anhed.	12.2	(7.4)	9.4	(6.2)	*p* = 0.149
*Clinical Characteristics*					
BPRS Avg. Item Score	1.88	(0.32)			
SANS Avg. Item Score	1.57	(0.82)			
Antipsychotic Medications					
-Clozapine	N =	10			
-Risperidone	N =	8			
-Olanzapine	N =	5			
-Quetiapine	N =	2			
-Ziprasidone	N =	1			
-Risp + Olanz	N =	2			

Abbreviations: Diff., difference; ns, non-significant; F, female; M, male; W, White; NW, non-White; WASI, Wechsler Abbreviated Scale of Intelligence; WTAR, Wechsler Test of Adult Reading; RBANS, Repeatable Battery for the Assessment of Neuropsychological Status; Phys., physical; Anhed., anhedonia; Soc., social; BPRS, Brief Psychiatric Rating Scale; SANS, Scale for the Assessment of Negative Symptoms; Risp, risperidone; Olanz, olanzapine. Numbers in parentheses are standard deviations. Note: two SZ patients did not complete the Chapman scales. Bold *p*-values indicate statistical significance at *p* < 0.05.

## Data Availability

The data presented in this study are available on request from the corresponding author.

## Supplementary Materials

The following are available online at https://www.mdpi.com/article/10.3390/brainsci11121610/s1. Table S1: Neuroimaging Contrast Descriptions, Table S2: Regions-of-Interest Locations, Table S3: Characterizing Data for Participants in the Reduced Sample, Figure S1: Reduced Sample Behavioral Results, Figure S2: Ventral Striatal Response to Prediction Error in Reduced Sample, Figure S3: BOLD activation of Salience Nodes to fMRI contrasts in Reduced Sample, Figure S4: BOLD activation of Salience Nodes to fMRI contrasts in Reduced Sample, Figure S5: Association between DMN node and Positive Symptom Severity in Reduced Sample, Figure S6: Correlation Matrix between Salience Nodes and Negative Symptom Severity, Figure S7: Correlation Matrix between Salience Nodes and Positive Symptom Severity, Figure S8: Correlation Matrix between DMN Nodes and Negative Symptom Severity, Figure S9: Correlation Matrix between DMN Nodes and Positive Symptom Severity.
