# Supplementary material for: Schizophrenia Patients Show Largely Similar Salience Signaling Compared to Healthy Controls in an Observational Task Environment
Source: Brain Sci. 2021 Dec 6;11(12):1610. doi: 10.3390/brainsci11121610 (PMC8699423; doi:10.3390/brainsci11121610)
Supplement: Supplementary file 1 [file brainsci-11-01610-s001.zip › brainsci-1472673-supplementary.pdf]

Supplemental Materials for

Do schizophrenia patients show aberrant salience signaling in observational environments?

Adam J. Culbreth[1]
Zuzana Kasanova[2]
Thomas J. Ross[3]
Betty J. Salmeron[3]
James M. Gold[1]
Elliot A. Stein[3]
James A. Waltz[1]

1. Maryland Psychiatric Research Center, Department of Psychiatry, University of Maryland School of Medicine, Baltimore, MD, USA
2. Center for Contextual Psychiatry, KU Leuven, Kapucijnenvoer 33 bus 7001, 3000 Leuven, Belgium
3. Neuroimaging Research Branch, National Institute on Drug Abuse - Intramural Research Program, Baltimore, MD, USA

Supplementary Methods: 118 words

Supplementary Results: 598 words

Supplementary Tables

1. Neuroimaging Contrast Descriptions
2. Regions-of-Interest Locations
3. Characterizing Data for participants in the reduced sample

Supplementary Figures

1. Reduced Sample Behavioral Results
2. Ventral Striatal Response to Prediction Error in Reduced Sample
3. BOLD activation of Salience Nodes to fMRI contrasts in Reduced Sample
4. BOLD activation of Salience Nodes to fMRI contrasts in Reduced Sample
5. Association between DMN node and Positive Symptom Severity in Reduced Sample
6. Correlation Matrix between Salience Nodes and Negative Symptom Severity
7. Correlation Matrix between Salience Nodes and Positive Symptom Severity
8. Correlation Matrix between DMN Nodes and Negative Symptom Severity
9. Correlation Matrix between DMN Nodes and Positive Symptom Severity

Supplementary Methods

Reduced Sample Characterizing Data

As stated in the manuscript, we conducted a supplementary analysis of the MRI data to rule out a general performance deficit as an explanation for differences in brain activations. Thus, in a second set of group-analyses of MRI data identical to the analyses mentioned above, we included only participants who demonstrated sufficient acquisition of the relative reinforcement probabilities of the four cards. Subjects' whose average between-run reinforcement-frequency estimates were not within 20% of actual contingencies were excluded. These criteria resulted in the removal of 11 patients and 3 controls from the initial analyses, leaving 17 patients and 20 controls. Characterizing information for participants included in the reduced sample is shown in Supplementary Table S1.

Supplementary Results

Behavioral Results

As shown in Panels C and D of Figure S1 (Panels A and B show results in the full sample for comparison), in the reduced sample both groups showed learning of reward probabilities and sensitivity to contingencies associated with particular cues. Specifically, participants: 1) modulated their trial-wise predictions of winning/losing, given the reward probabilities associated with particular cues; 2) self-reported appropriate estimates of the reward probabilities for particular cues when asked between runs. A two-way ANOVA for trial-by-trial reward predictions, with factors of GROUP and CUE, revealed a significant main effect of CUE [$F(3,35)=204.97$, $p<0.001$], but no main effect of GROUP [$F(1,35)=1.20$, $p=0.28$] or GROUP x CUE interaction [$F(1,35)=1.75$, $p=0.16$]. The two-way ANOVA for estimated reward frequency, with factors of GROUP and CUE, revealed a significant main effect of CUE [$F(1,23)=239.99$, $p<0.001$], but no main effect of GROUP [$F(1,23)=0.59$, $p=0.45$] or GROUP x CUE interaction [$F(1,35)=1.08$, $p=0.35$]. Thus, in terms of behavior, both the reduced and full

samples showed robust effects of CUE; however, the small effects of GROUP and GROUP X CUE interactions observed in the full sample were not found in the reduced sample.

MRI Results

Regions-of-Interest Analyses

Analyses of PE-signaling in ventral striatum. As shown in Figure 3, we observed robust deactivations to unexpected reward omissions in the entire sample in both left and right VS, and the groups did not differ in the magnitudes of these deactivations in either left or right VS. We did *not* observe robust *activations* to unexpected reward deliveries in the entire sample in either left or right VS. These findings were similar when examined in the whole sample (Figure S2).

Salience Network nodes. In the reduced sample, across all participants the anterior insula showed significantly greater BOLD activation for greater prediction error magnitude than less [Contrast 3 in Table S1; Left AI: $t(36) = 1.97$, $p = 0.06$; Right AI: $t(36) = 3.05$, $p = 0.004$] and greater reward omission likelihood than less [Contrast 5; Left AI: $t(36) = 2.56$, $p = 0.02$; Right AI: $t(36) = 2.7$, $p = 0.01$], but not greater uncertainty vs. less [Contrast 6; Left AI: $t(50) = 0.70$, $p = 0.49$; Right AI: $t(36) = 1.51$, $p = 0.14$; Figure S3]. Similarly, significantly greater activations were observed in right inferior and superior parietal ROIs for greater prediction error magnitude vs. less [Right Superior Parietal: $t(36) = 2.86$, $p = 0.007$; Right Inferior Parietal: $t(36) = 1.84$, $p = 0.07$] and greater uncertainty vs. less [Right Superior Parietal: $t(36) = 2.8$, $p = 0.008$; Right Inferior Parietal: $t(36) = 2.43$, $p = 0.02$]. As with the analyses of the full sample, we (surprisingly) observed no significant between-group differences in BOLD signal contrasts for different types of salience.

DMN Nodes. In the reduced sample, across all participants, multiple ROIs within the DMN were reliably deactivated for greater uncertainty. Specifically, greater deactivation was observed in both left supramarginal gyrus [t(36) = -3.3, p = 0.002] and left superior frontal gyrus [t(36) = -2.3, p = 0.03; Figure S4]. The effect of uncertainty on right medial frontal gyrus BOLD activation was in the same direction as the full sample but failed to reach significance [t(36) = -1.68, p = 0.1]. No other significant effects were noted within the DMN for the other salience contrasts (i.e., prediction error valence, prediction error magnitude, likely reward omission). Further, we did not observe significant between-group differences in any salience contrasts in any DMN nodes, in the reduced sample.

Correlation analyses between ROI activation and symptom severity. Similar to the full sample, positive symptom severity (BPRS reality distortion) in those with schizophrenia was positively associated with BOLD activation in DMN nodes for the prediction error valence, including the left supramarginal gyrus (r = 0.68, p = 0.003; Figure S5). No other significant symptom effects were identified (see figures S6-S9 for a full presentation of correlational analyses).

Table S1. Neuroimaging Contrast Descriptions.

Contrast Index	Contrast Name	Description of Contrast	Cues Involved in Contrast
1	Positive PE	brain responses to unexpected reward deliveries	(20W + 40W) - (20N - 40N)
2	Negative PE	brain responses to unexpected reward omissions	(60N + 80N) - (60W + 80W)
3	PE Magnitude	brain responses to unexpected outcomes	(20W + 40W + 60N + 80N) - (20N + 40N + 60W + 80W)
4	PE Valence	brain responses to obtained gains	(20W + 40W + 60W + 80W) - (20N + 40N + 60N + 80N)
5	Punish Likelihood Effect	brain responses to cues predictive of no gain	(60W + 80W + 60N + 80N) - (20W + 40W + 20N + 40N)
6	Uncertainty Effect	brain responses to cues associated with uncertain outcomes	(40W + 60W + 40N + 60N) - (20W + 80W + 20N + 80N)

Abbreviations: PE, Prediction Effort; W, Win Trial; N, No-win Trial.

Table S2. Regions-of-Interest Locations

Region	MNI Coordinates (x, y, z)	Reference
Salience		
Left Anterior Insula	(-28,19,4)	Huettel et al., 2005
Right Anterior Insula	(37, 26, 4)	Huettel et al., 2005
Left Superior Parietal Lobule	(-12,-75,44)	Huettel et al., 2005
Right Superior Parietal Lobule	(29, -67, 44)	Huettel et al., 2005
Left Inferior Parietal Lobule	(-22, -58, 44)	Huettel et al., 2005
Right Inferior Parietal Lobule	(43, -46, 44)	Huettel et al., 2005
Default Mode Network		
Left Medial Prefrontal Cortex	(-21, 63, 18)	Waltz et al., 2013
Right Medial Prefrontal Cortex	(10, 58, 8)	Waltz et al., 2013
Left Superior Frontal Gyrus	(-29, 19, 47)	Waltz et al., 2013
Right Superior Frontal Gyrus	(23, 26, 49)	Waltz et al., 2013
Left Supramarginal Gyrus	(-46, -64, 32)	Waltz et al., 2013
Right Supramarginal Gyrus	(48, -60, 32)	Waltz et al., 2013
Posterior Cingulate Cortex	(-1, -52, 34)	Waltz et al., 2013
Reward		
Right Ventral Striatum	(+10, 8, -4)	Pessiglione et al., 2005
Left Ventral Striatum	(-10, 8, -4)	Pessiglione et al., 2005

Table S3. Characterizing Data for Participants in the Reduced Sample

	Patients	(N=17)	Controls	(N=20)	Sig. of Group Diff.
Demographics					
Age	38.3	(10.8)	40.2	(11.1)	p = 0.611
Gender	4 F,	13 M	6 F,	14 M	p = 0.725
Race	13 W,	4 NW	14 W,	6 NW	p = 0.725
Smokers	6 Y,	11 N	5 Y,	15 N	p = 0.719
Subject Education (years)	13.7	(1.8)	15.3	(2.1)	p = 0.015
Parental Education (years)	14.6	(2.3)	14.2	(2.3)	p = 0.592
Neuropsychological Testing/ Questionnaires					
IQ (from WASI 4-subtest)	107.4	(14.7)	116.4	(12.4)	p = 0.052
WTAR Scaled Score	107.6	(13.5)	109.7	(12.1)	p = 0.618
RBANS Total	92.7	(15.8)	103.5	(9.6)	p = 0.015
Chapman – Phys. Anhed.	12.8	(4.7)	11.0	(9.6)	p = 0.500
Chapman – Soc. Anhed.	10.3	(6.3)	9.6	(6.8)	p = 0.765
Clinical Characteristics					
Mean BPRS Item Score	1.7	(0.3)			
Mean SANS Global Item Score	1.6	(0.8)			
Antipsychotic Medications					
- Clozapine	N=	6			
- Risperidone	N=	6			
- Olanzapine	N=	2			
- Quetiapine	N=	1			
- Ziprasidone	N=	1			
- Risp+Olanz	N=	1			
Mean APD dose*	8.9	(6.8)			

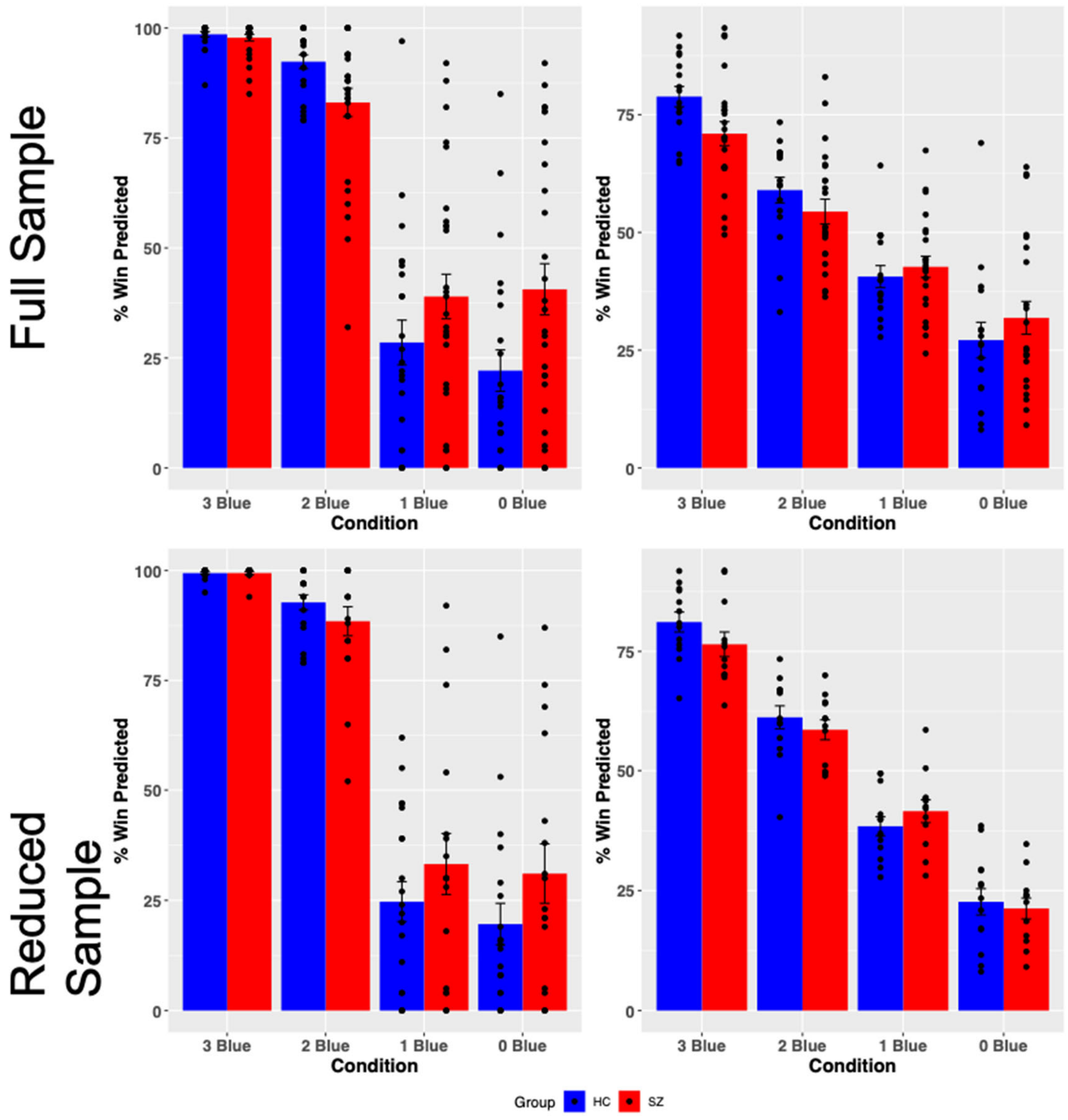

Figure S1 Reduced Sample Behavioral Results: Participants: 1) modulated their trial-wise predictions of winning/losing, given the reward probabilities associated with particular cues (S1 A for full sample; S1 C for reduced sample); 2) self-reported appropriate estimates of the reward probabilities for particular cues when asked between runs (S1 B for full sample; S1 D for reduced sample).

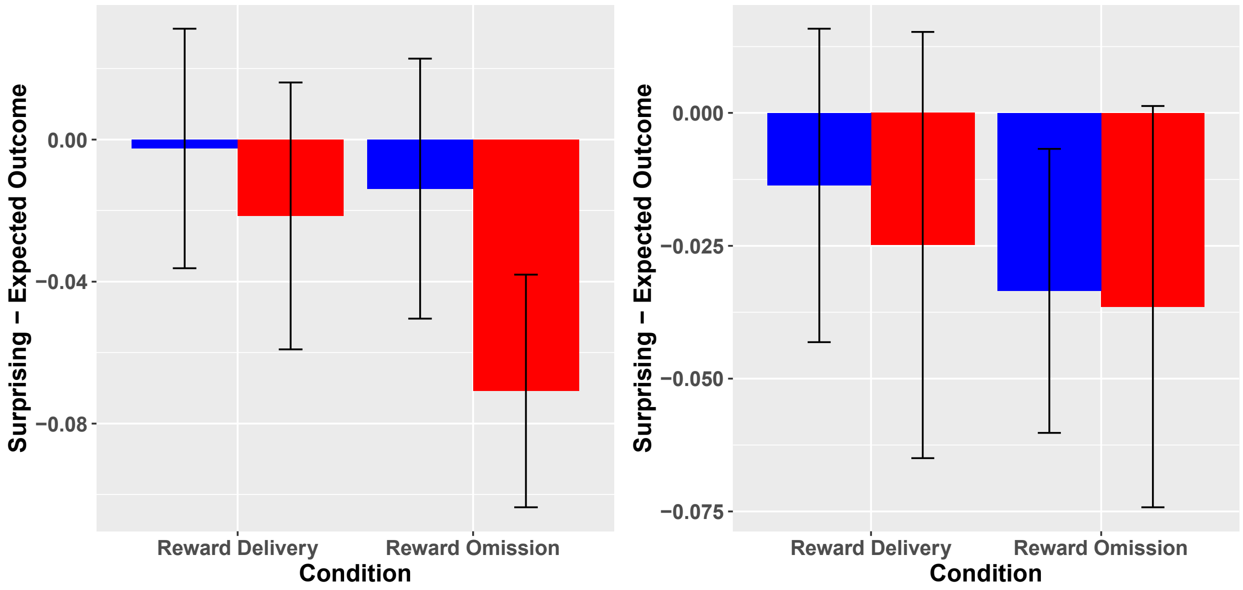

Figure S2: Ventral Striatal Response to Prediction Error in Reduced Sample: Similar to the full sample, Robust signaling of surprising reward omission, but not surprising reward deliveries, is evidence in both right (right image) and left (left image) ventral striatum for the reduced sample. No group differences were observed.

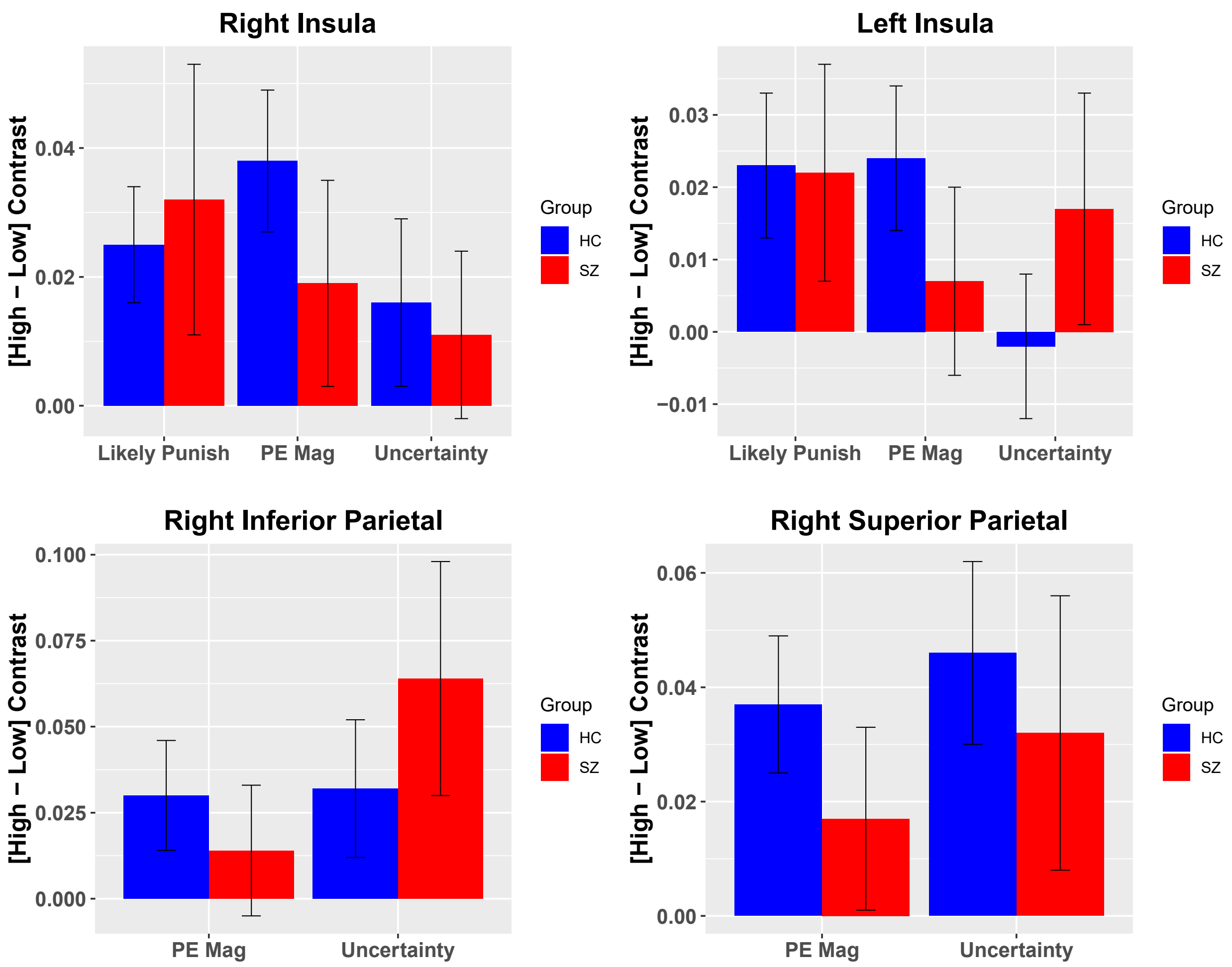

Figure S3: BOLD activation of Salience Nodes to fMRI contrasts in Reduced Sample: error bars represent standard error of the mean; HC = Healthy Control, SZ = Schizophrenia.

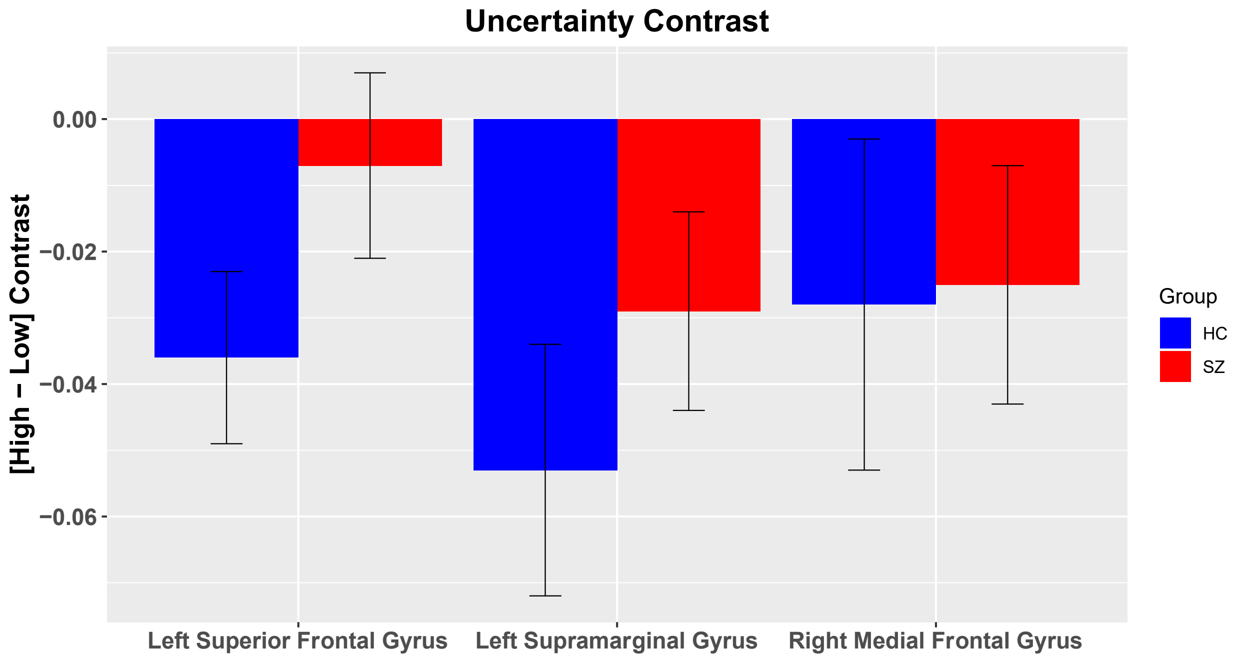

Figure S4: BOLD activation of Salience Nodes to fMRI contrasts in Reduced Sample: error bars represent standard error of the mean; HC = Healthy Control, SZ = Schizophrenia.

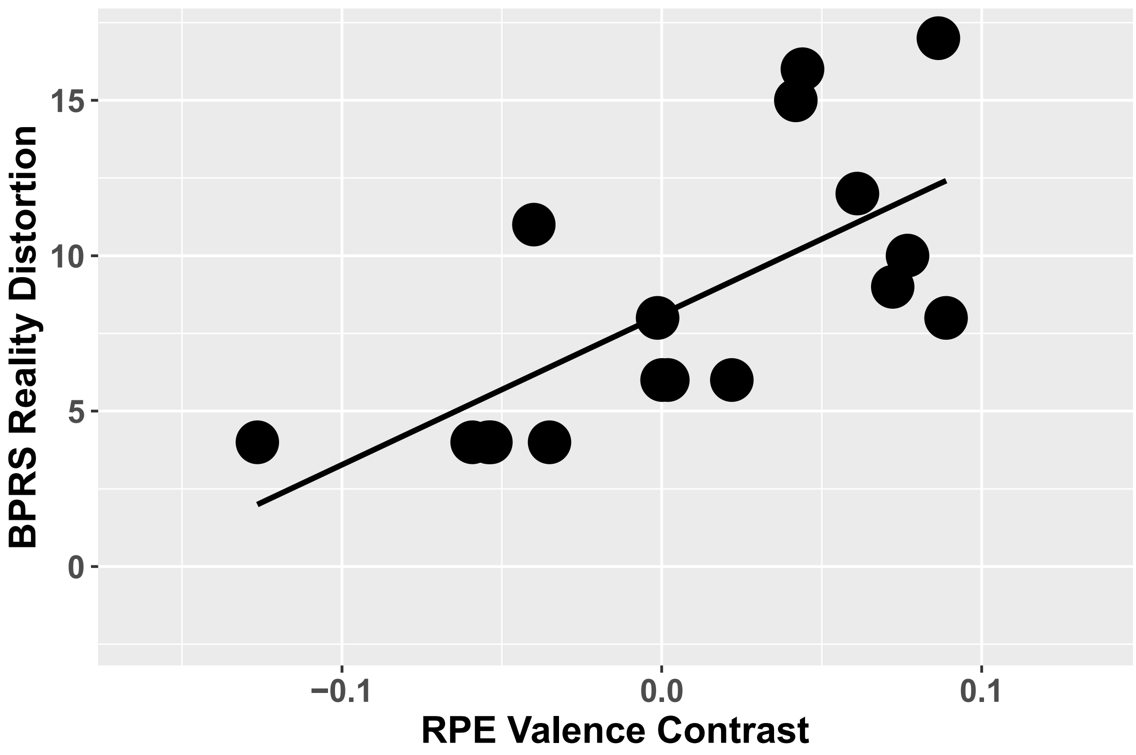

Figure S5: Association between DMN node and Positive Symptom Severity in Reduced Sample

Full Sample

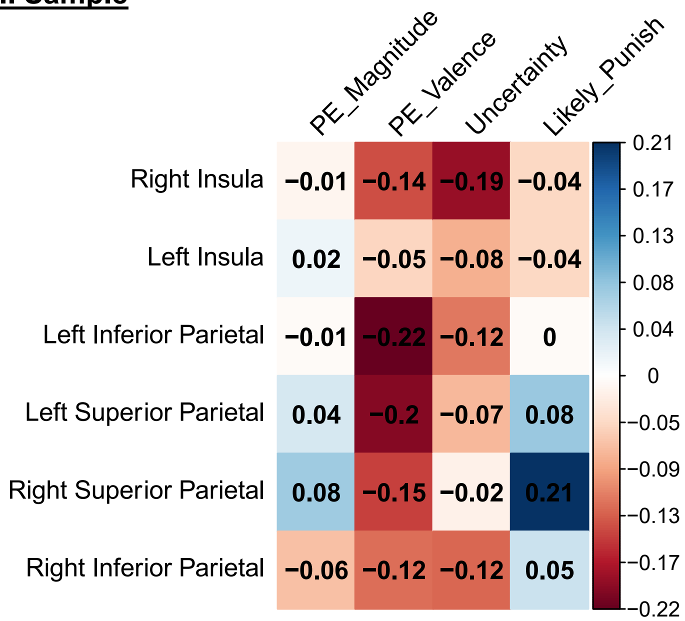

Reduced Sample

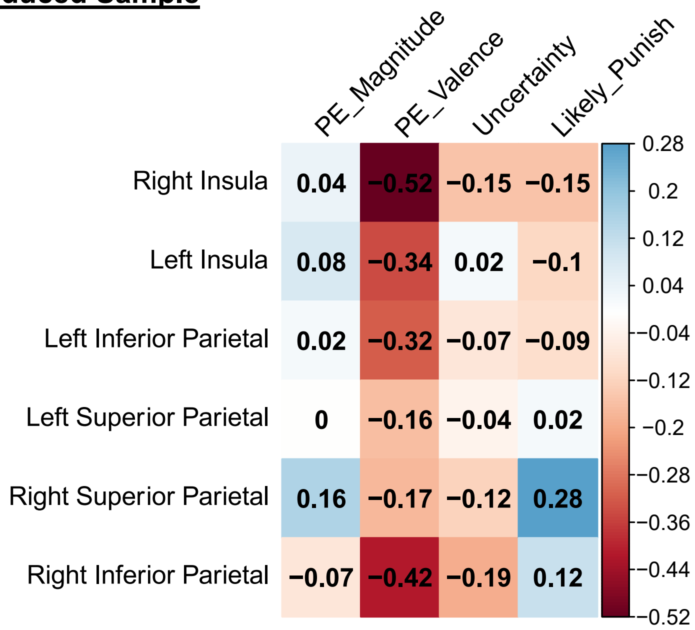

Figure S6: Correlation Matrix between Salience Nodes and Negative Symptom Severity

Full Sample

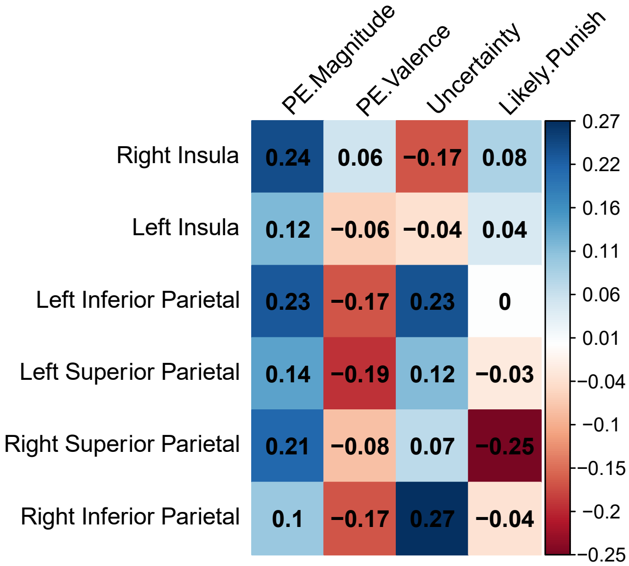

Reduced Sample

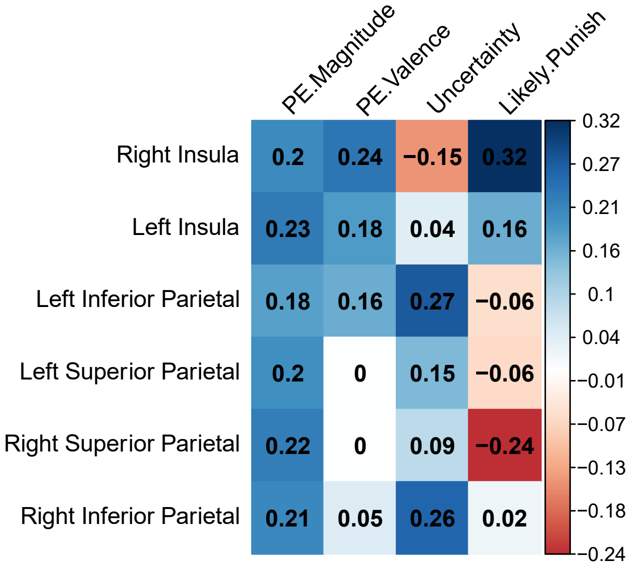

Figure S7: Correlation Matrix between Salience Nodes and Positive Symptom Severity

Full Sample

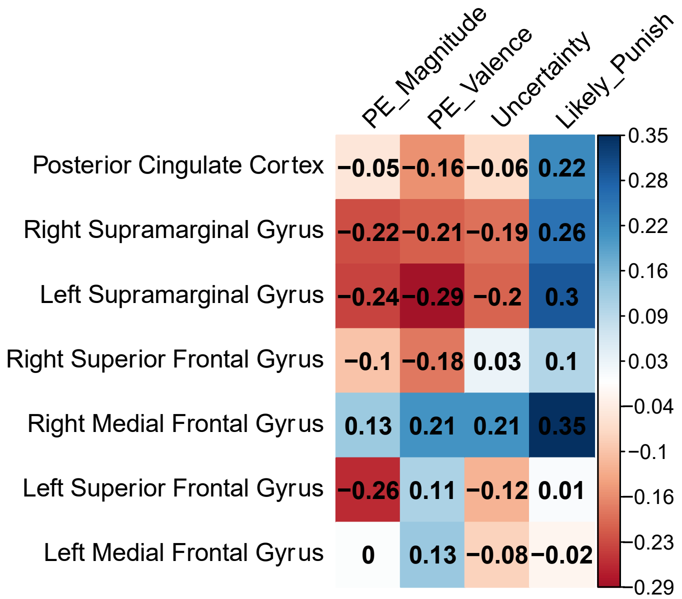

Reduced Sample

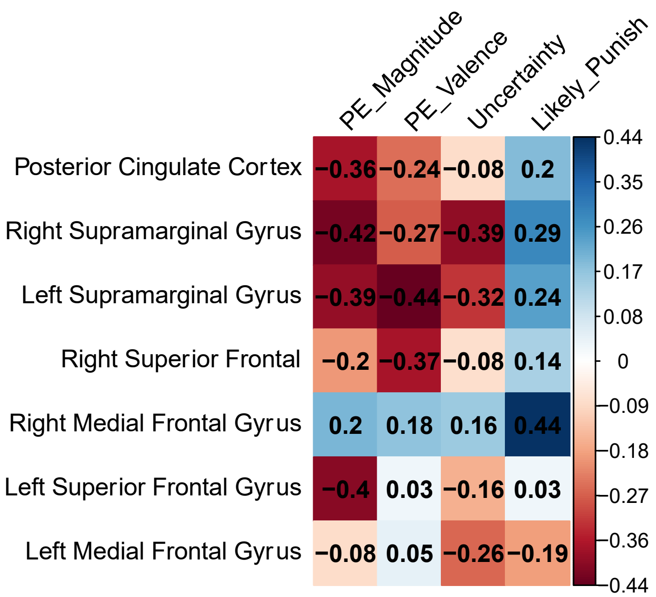

Figure S8: Correlation Matrix between DMN Nodes and Negative Symptom Severity

Full Sample

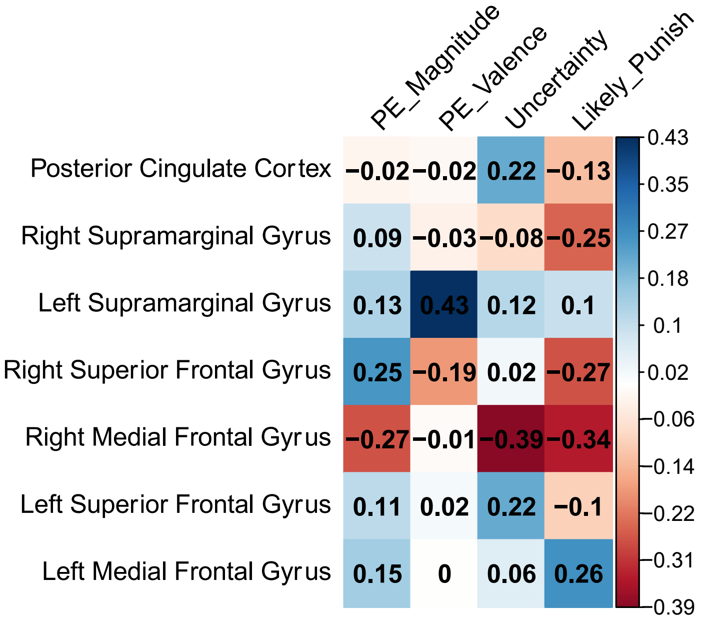

Reduced Sample

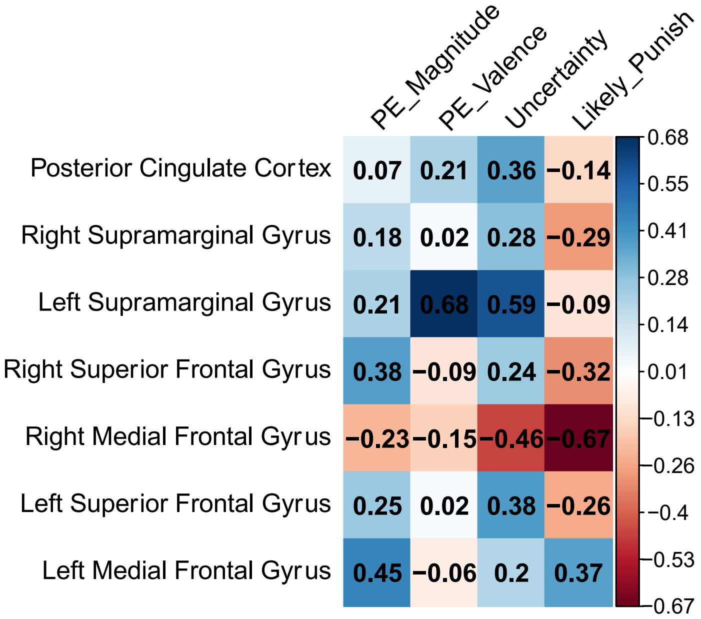

Figure S9: Correlation Matrix between DMN Nodes and Positive Symptom Severity
